# Availability of antimalarial medicines and inventory management at the community level: a case study of Bugesera district in Rwanda

**DOI:** 10.1186/s12913-024-10605-z

**Published:** 2024-01-24

**Authors:** Godelive Umulerwa Gakinahe, Eugene Rutungwa, Francois Mbonyinshuti, Egide Kayitare

**Affiliations:** 1 EAC Regional Centre of Excellence for Vaccines, Immunization and Health Supply Chain Management, College of Medicine and Health Sciences, University of Rwanda, Kigali, Rwanda; 2 School of Business, College of Business and Economics, University of Rwanda, Kigali, Rwanda; 3 Ministry of Health, Kigali, Rwanda; 4 School of Medicine and Pharmacy, College of Medicine and health sciences, University of Rwanda, Kigali, Rwanda

**Keywords:** Availability, Antimalarial medicines, Inventory management, Bugesera, Rwanda

## Abstract

**Background:**

Malaria is a public health hazard globally, with Sub-Saharan Africa accounting for more than 90% of malaria deaths, primarily affecting children under the age of five. In Rwanda, malaria interventions include the availability of antimalarial medications, notably Artemisinin-based combination treatments (ACTs) and quick diagnostic test kits (RDTs). However, the availability of antimalarial medicines and its related inventory management at community level in Rwanda has yet to be identified.

**Methods:**

The study was conducted using a descriptive cross-sectional research design. The study involved the Community Health Workers (CHWs) in Bugesera District, working as a team of one male-female pair called *Binômes*. CHWs provide Integrated Community Case Management (iCCM) and treatment of Malaria in the villages. The sample size was 295 and respondents were selected using convenient random sampling from 15 sectors of Bugesera District, each cell being represented. A structured research questionnaire was used to collect data. The questionnaires were filled by CHWs who were available for this study at the time of data collection. Collected data were exported to SPSS version 26 for coding and analysis.

**Results:**

The CHWs reported to be actively involved in managing the antimalarial medicines inventory. Overall, 64.1% of CHWs indicated that the population could easily obtain antimalarial medicines from community health workers and 31.2% attested that people could also obtain antimalarial medicines from the health centers. Majority of respondents 78% confirmed that the CHWs had the appropriate storage conditions for antimalarial medicines, while the overall stock out recorded was 3.20%. Furthermore, CHWs described some challenges, including but not limited to, lack of appropriate or dependable transportation and inappropriate medicines storage. Transportation was reported as a critical barrier for accessing antimalarial medicines. The majority, 70,85% travel on foot while 25.4% reported that CHWs walk for a distance between 1 and 2 h for resupply of antimalarial medicines.

**Conclusion:**

This study investigated the availability of antimalarial medicines and inventory management challenges at community level in Rwanda. Addressing these challenges will reduce the rate of stockout and increase the availability of antimalarial medicine at community level. Appropriate storage, and reduction of stock out rate, will serve to strengthen the current CHWs system, and provide critical guidance for the evolution of CHWs’ systems in Rwanda.

**Supplementary Information:**

The online version contains supplementary material available at 10.1186/s12913-024-10605-z.

## Background

Malaria is one of the world’s top causes of mortality in tropical and subtropical area [1]. More than 90% of malaria deaths worldwide occur in sub-Saharan Africa, affecting mainly children under five years of age, who bear 78% of the burden of all malaria-related death [2]. In addition, Malaria is shown to be a leading cause of anemia among pregnant women with low birth weight, premature, fetal, and perinatal mortality, and anemia-related maternal mortality [3].

Rwanda, among other sub- Saharan Africa countries, recorded a drop in malaria incidence from 400 per 1000 in 2016 to 148 per 1000 in 2020, Malaria-related deaths decreased for the same period from 700 to 148 deaths [4]. This was due to several interventions that Rwanda has leveraged to control malaria endemic.

Interventions which contributed to the decrease of malaria deaths include Indoor Residual Spraying (IRS) in high malaria burden districts, deploying Long-Lasing Insecticidal Nets (LLINs), and scalp up of home- based management of malaria to all ages and all districts, among others [5]. Currently, 56% of all malaria cases are managed by CHWs at home [4].

In October 2006, Rwanda rolled out artemisinin-based combination therapy (ACT) countrywide at the health center level [6]. In 2007, the use of ACTs for the treatment of uncomplicated Malaria was extended to the community level through a network of 30,000 community health workers [4].

Rwanda has four referral hospitals, 42 district hospitals, 438 health centers, and 45,011 CHWs operating in 14,873 villages. All CHWs are organized into Community Health Worker Cooperative. Each health center oversees one CHW cooperative [7].

Each village also has two multi-disciplinary CHWs (binômes: one man and one woman) who carry out:

i) Integrated community case management, or ICCM (assessment, classification and treatment or referral of diarrhea, pneumonia, malaria, and malnutrition in children under five years of age). ii) Malnutrition screening iii) Community-based provision of contraceptives iv) Preventive NCDs v) Prevention and behavior change activities vi) Household visits [7].

In recent years, the Rwandan Ministry of Health worked closely with implementing partners including USAID and PEPFAR to strengthen the national supply chain management (SCM) system to ensure that health products are continuously available to the people who need them. Community health workers (CHWs) are the last mile of the supply chain and the most difficult to reach with life-saving medications and supplies.

**Fig. 1:**
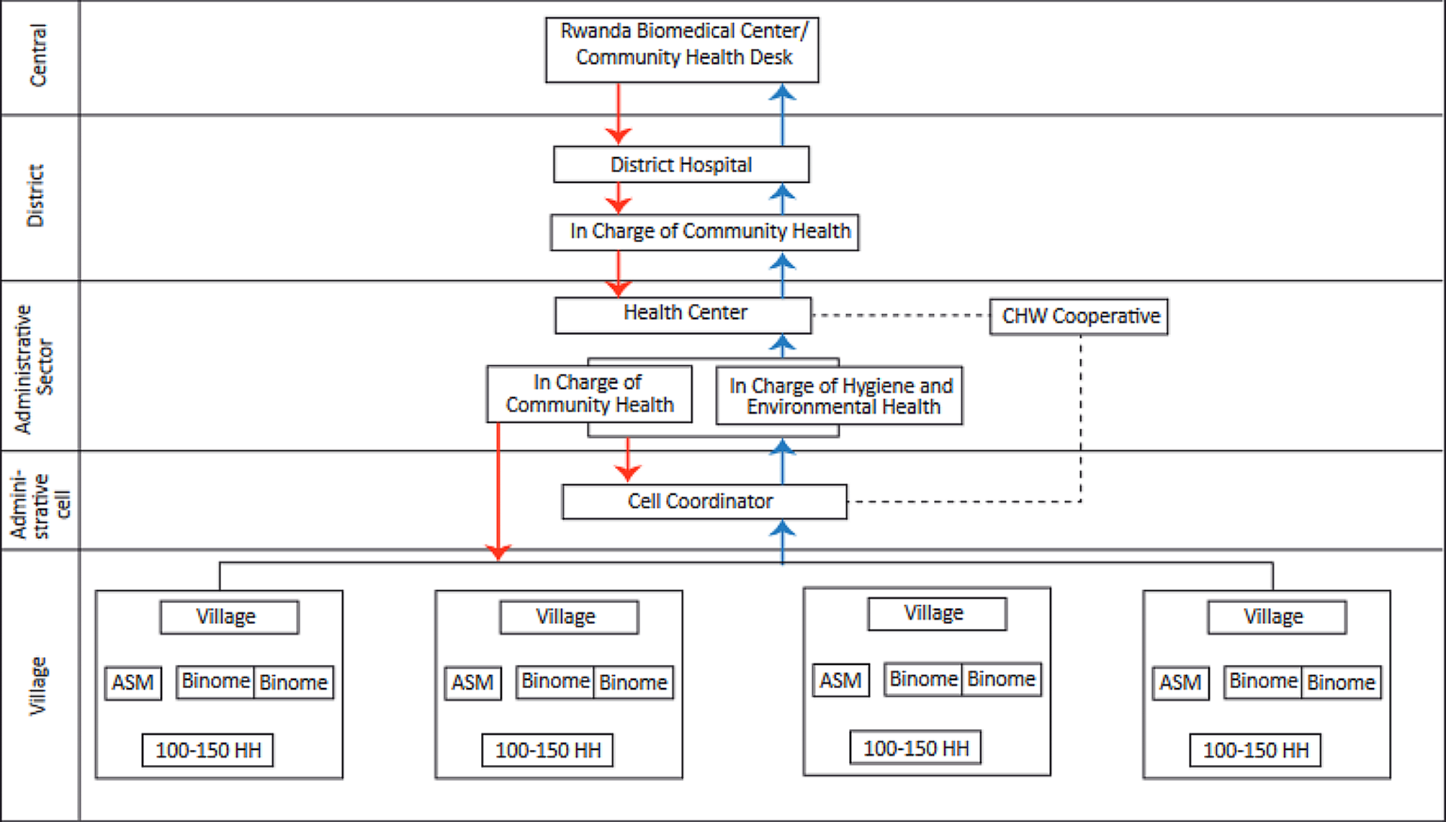
Linkage between the level of the Rwandan Health system and the CHW

Figure 1 indicates the linkage between the level of the Rwandan Health system and the CHW.

The Community Health Program Investment Case in Rwanda of August 2021 provides the linkage between different Stakeholders at different levels of the health system (central, district, sector, and community) to ensure the CHP is effective in its mission to deliver health services to the population. CHWs are elected by members of their communities based on ten-point criteria outlined below: (i) being able to read, write and calculate (ii) Being appreciated as honest by community peers (iii) Willing to maintain confidentiality (iv) Accepting volunteer status (v) Being a resident of the village (vi) having age between 20 and 50 years (vii) being available and accessible person (viii) not occupying a position of local leader (ix) being exemplary and serve as a positive role (x) as well as being elected by the community [8].

After selection, the CHWs receive trainings. Over the years, whenever a new CHW service package was added, the Rwanda Ministry of Health developed for them requisite resources including treatment protocols, guidelines, reporting templates and registers. These resources are used to train CHWs according to their roles and enables them to provide services of desired quality. The content of the training depends on the area of CHWs program component (Family Planning, ICCM etc.) and the scope of practices. Among others, the CHWs got training on how to use Rapid Test Diagnosis (RTDs) for distinguishing between malaria-induced and non-malaria-induced fevers and subsequently how to treat fever appropriately. In addition, trainings empower CHWs to identify and manage illnesses; or to refer to the health center for more professional treatment patients that they cannot manage [8].

Community Health Workers play a vital role in translating the national strategy into practice by providing information and resources for prompt and effective malaria treatment and prevention in local communities [9].

Community Health Workers primarily implement the health supply chain system, ensuring an effective and efficient distribution system for health products at the community level [10]. Different health providers are involved in the management of health commodities and these include CHWs [11]. The service they provide is saving life and have remarkable impact in the community’s health. In addition, drug availability is an essential component of CHW’s motivation, while the lack of drugs has been shown to decrease their morale and the community’s perception of their effectiveness [12]. However, the community health workers often face lack of supply chain management skills in general, and inventory management system in particular [13]. Thus, this affects the quality service delivery to the community in the management of health commodities in general, and the antimalarial products, in particular. Therefore, this study aimed to assess the availability of antimalarial medicines and the inventory management at the community level in Rwanda considering Bugesera District as a case study.

## Main research objective

The main objective of this research was to evaluate the availability of antimalarial medicines and the inventory management at the community level in Bugesera District.

### Specific objectives

1. Assess the availability and stockout level of antimalarial medicines at the community level in Bugesera District.
2. Identify the inventory management practices for antimalarial medicines at the community level in Bugesera District.
3. Identify prevailing challenges in inventory management practices of antimalarial medicines at the community level in Bugesera District.

### Research Questions

1. What is the availability and rate of stockout of antimalarial medicines at the community level in Bugesera District?
2. What are the inventory management practices for antimalarial medicines at the community level in Bugesera District?
3. What are the challenges in inventory management practices of antimalarial medicines at the community level in Bugesera District?

## Methods

### Study setting

This study was conducted in Bugesera District, Eastern province in Rwanda. According to the fourth Rwanda Population Housing Census 2012, Bugesera District covers 1,337 km2. The district comprises of 15 sectors, 72 cells, and 581 villages with a total Community Health Workers of 1,132. Moreover, according to the MOPDD’s health management information system (HMIS), data from 2016 classified Bugesera as one of the five high-burden districts in Rwanda, accounting for over 70% of the disease burden [14].

### Study design and population

The study adopted a descriptive, cross-sectional research design using structured questionnaires to capture the required data among community health workers in Bugesera district of Eastern Province. Data were related to the consumptions of November 2021 which were collected from December 2021 to January 2022. Community Health Workers in Bugesera District were selected from CHWs called *Binômes*, who are in charge of community case management, and treatment of Malaria at the village level.

### Sampling

For the study population, researchers selected community Health Workers in Bugesera District, precisely one male-female pair of CHWs called Binômes, who provide iCCM and treatment of Malaria in a village. Bugesera District counts a total number of 1132 CHWs. The sample size for study was calculated using Taro Yamane formula [15]. A sample of 295 CHWs was selected by convenient random sampling and each cell in Bugesera District was represented by minimum 4 participants.

### Data collection tool and methods

Data were collected using a structured questionnaire adapted from the Assessment of Malaria Pharmaceutical Management Systems in Ghana authored by Arhinful et al. [16]. Participants were selected from the list provided by the Head of CHWs cooperatives. The Head of cooperatives provided the CHWs telephone numbers used to request for an appointment of visit; if phone call was unsuccessful, after three attempts, data collector replaced the CHW by another one available on the list. On the appointment date, the researcher visited the CHWs to complete the questionnaire at their home. To ensure data uniformity, research assistants were trained and involved in data collection.

### Data analysis

The collected data were checked for internal consistency and completeness, then entered and analyzed using SPSS Version 26. Findings were described and presented using frequencies and percentages for categorical variables and medians and Inter Quartile Range (IQR) for continuous variable. Also, data were presented using tables and figures.

## Results

The results of the study were presented and analyzed in detail according to the specific objectives of the study.

### Socio-demographic characteristics

The results, in Table 1, indicate that 53.9% respondents were women and 46.1% were men. The study revealed that 40.67% were in the age range of 35–45 years. While 57.6% had a primary education level, whereas 31.5% had secondary level.

**Table 1:** Socio demographics of the study participants

Variables	Characteristics	Frequency	Percentage
Gender	Female	159	53.9
	Male	136	46.1
Age group	25–35	90	30.5
	35–45	120	40.67
	45–55 Above 55	75 10	25.45 3.38
Level of education	Primary Secondary Higher education Others	170 93 4 28	57.6 31.5 1.4 9.5

### Consumption of artemether-lumefantrine (Coartem) for both young and adult doses in the community

This study found that a total of 1,825 blisters of Artemether-lumefantrine (Coartem) drugs, for all weight of ages were consumed in November 2021, as indicated in Table 2.

**Table 2:** Consumption of Artemether-lumefantrine (Coartem) for both young and adult doses in November 2021

Drug consumption of November 2021	N	Min.	Max.	Sum	Mean	Std. Deviation
1. Drug consumption for 5 to 15 kg	295	0	12	309	1.05	1.609
2. Drug consumption for 15 to 25 kg	295	0	12	378	1.28	1.747
3. Drug consumption for 25 to 35 kg	295	0	12	457	1.55	2.199
4. Drug consumption for above35 kg	295	0	17	681	2.31	2.839
Valid N (listwise)	295			1825		

### The rate of stock out of antimalarial medicines at the community level

The rate of stock out was calculated by considering the total number of stock-out days in November 2021 for all CHWs. Few CHWs recorded information on stock outs. The average of stock out rate, for all age range of Arthemether-lumefantrine was 3.20%, as indicated in Table 3.

**Table 3:** The rate of stock out of Artemether-lumefantrine (Coartem) for both young and adult doses

	N	Min (days)	Max (days)	Sum (days)	Mean (days)	Stock out rate
1. Stock out duration for 5–15 kg	295	0	60	312	1.06	3.52
2. Stock out duration for 15–25 kg	295	0	30	178	0.60	2.011
3. Stock out duration for 25-35 kg	295	0	90	386	1.31	4.36
3. Stock out duration for > 35 kg	295	0	30	260	0.88	2.93
Average						3.20

### The availability of appropriate medical storage at the community level

**Fig. 2:**
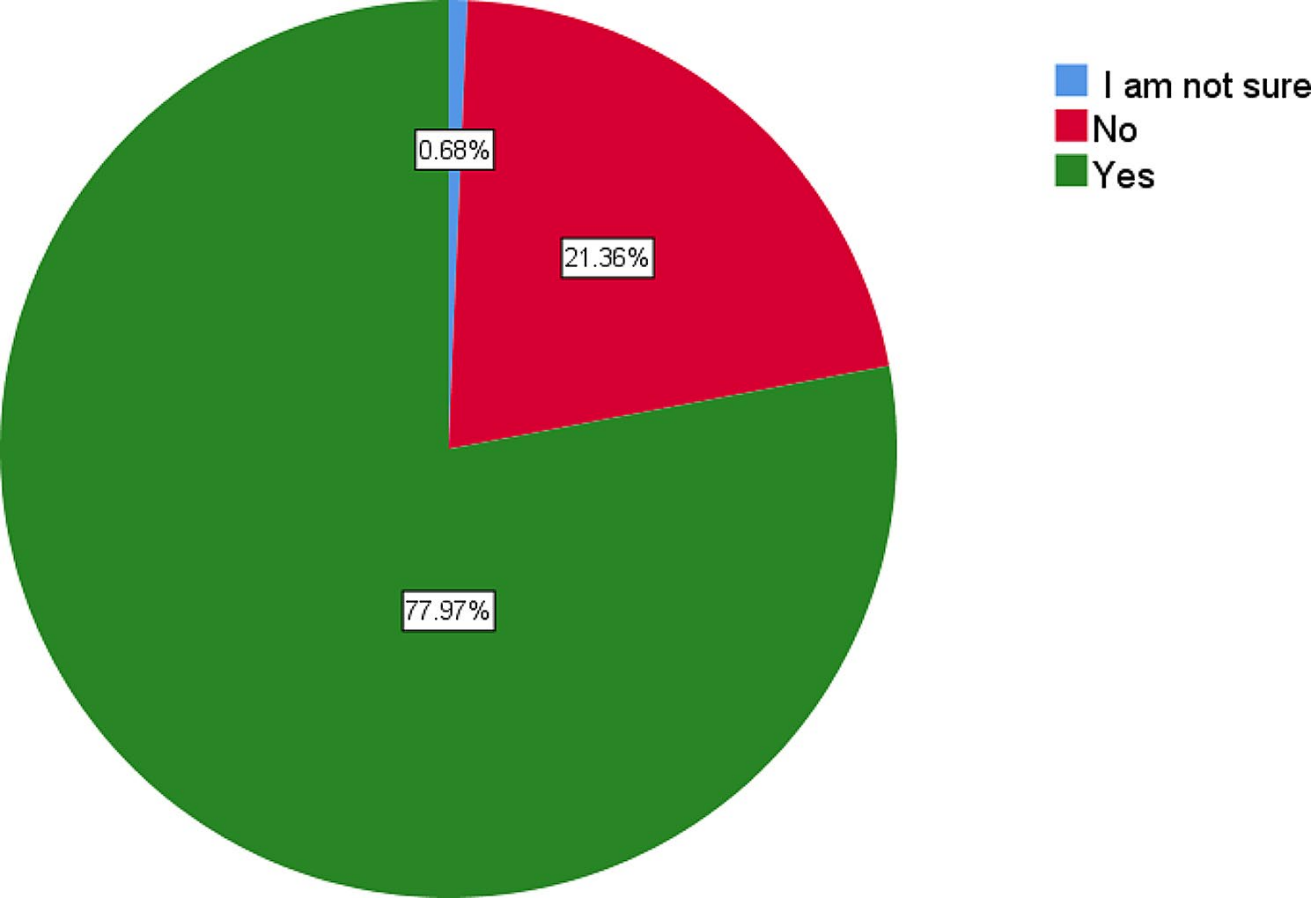
Appropriate medical storage at the community level

Majority of respondents, 77.97% reported to have an appropriate medical storage for antimalarial medicines in a safe storeroom, locked with key. Data collectors observed that most of the CHWs in Bugesera district stored antimalarial medicines in clean, dry, well-lit and well-ventilated storeroom, and away from walls as illustrated in Fig. 2.

### Availability of antimalarial medicines in the community level

**Fig. 3:**
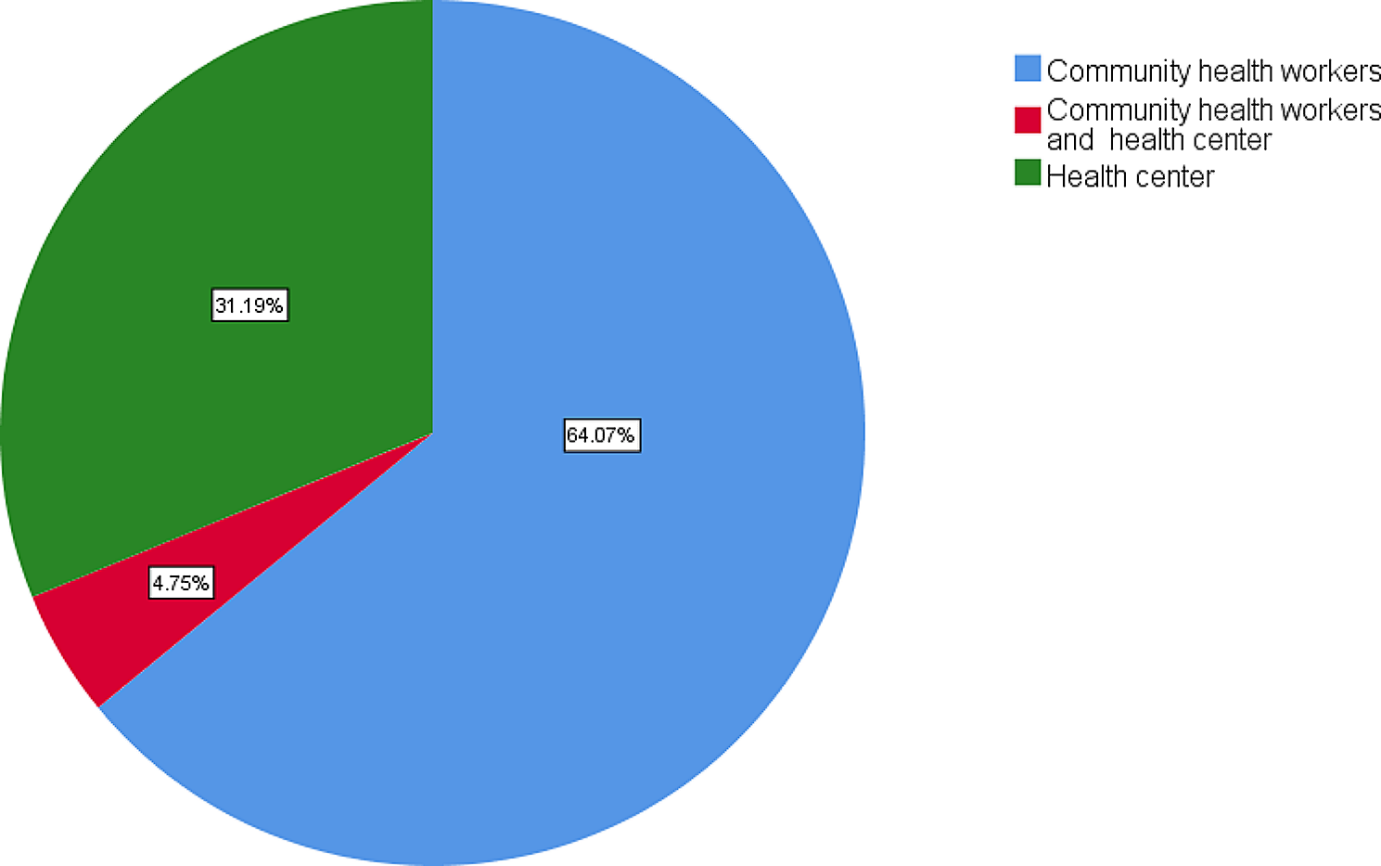
Availability of antimalarial medicines in the Community level

Respondents have confirmed that the antimalarial medicines are available at both health centers and community health workers. For the frequency of accessing antimalarial medicines in the community, majority of respondents, 64.07% of the CHWs stated that the population easily access the antimalarial medicines at the Community Health Workers. Respondents representing 31.19% agreed that people get antimalarial medicines from the health center as indicated in Fig. 3.

### Inventory management related challenges in distribution, reporting and transport of antimalarial medicines

#### Means of transport used by CHWs while collecting antimalarial medicines

**Fig. 4:**
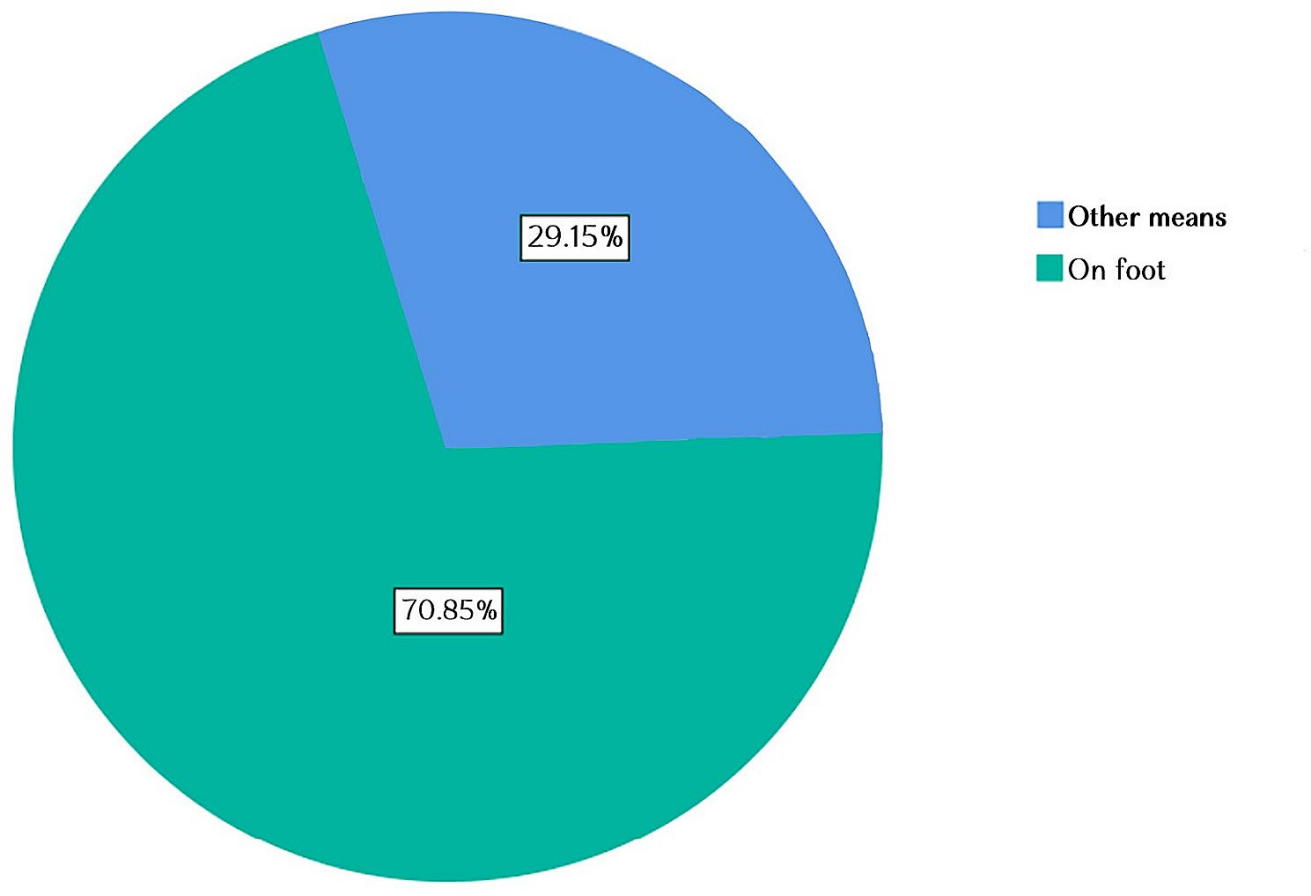
Means of transport used by community Health Workers

CHWs have reported lack of appropriate or dependable transportation as a critical barrier to accessing antimalarial medicines. As illustrated in Fig. 4, 70.85% of CHWs traveled on foot, while 29.15% traveled using other means of transport.

### The distance travelled by the CHWs for the resupply

**Fig. 5:**
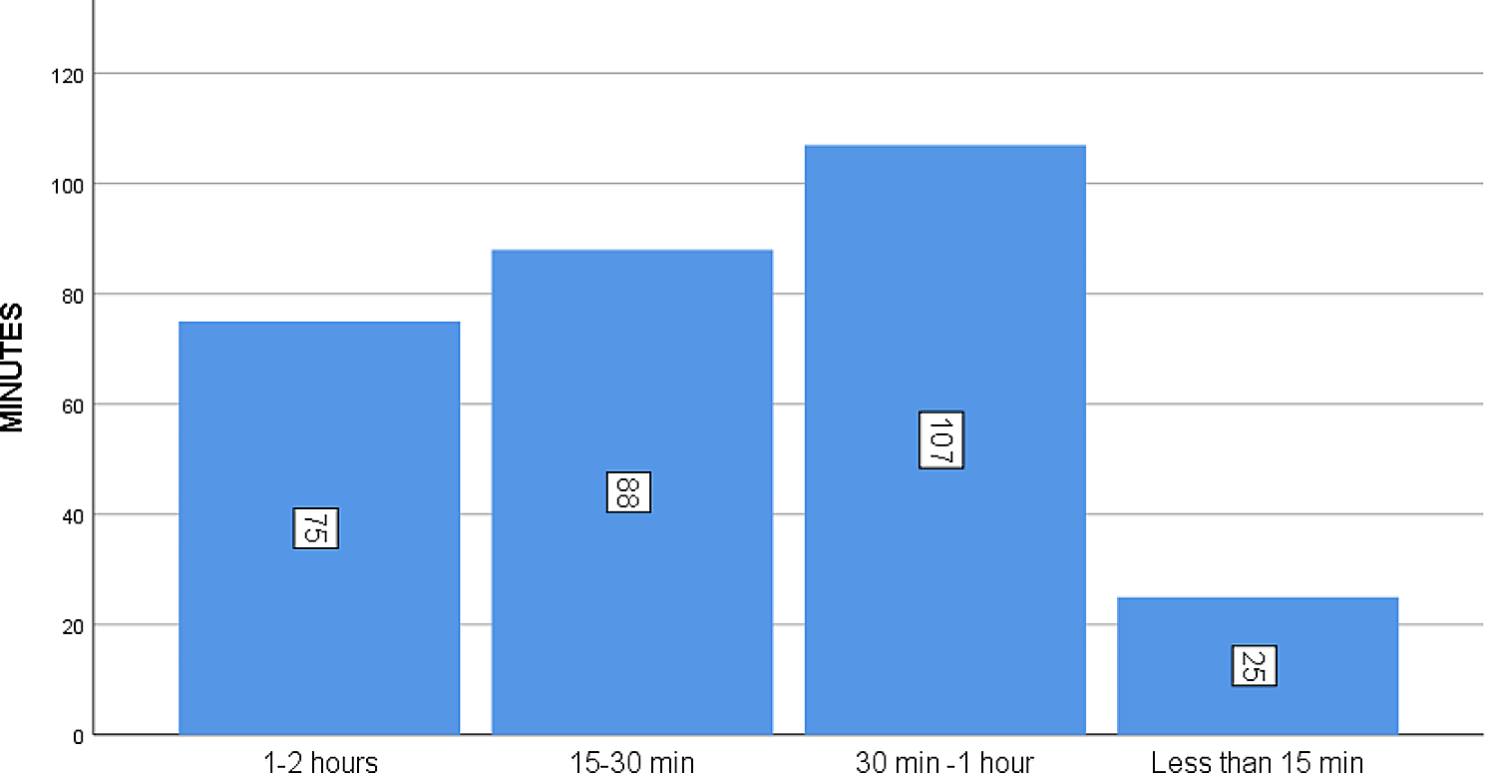
The distance travelled by the CHWs for the resupply

The distance travelled by the CHWs for the resupply was assessed, out of 295CHWs, 75 (25.4%) reported that they traveled the distance between 1 and 2 h while 107 (36.27%) indicated that they traiveled a distance between 30 min to one hour to resupply as indicated in Fig. 5.

### The amount of money CHWs spend from their own pocket

Overall, 51.2% CHWs stated that they spend more than 2,000 Rwandan Francs (RWF) per month from their own pocket on community health duties as transport as indicated in Table 4.

**Table 4:** The amount of money CHWs spend on their own pocket per month

		Frequency	Percent	Cumulative Percent
Valid	Less than 2000 RWF	64	21.7	21.7
	Greater than 2000 RWF	151	51.2	72.9
	Greater than 2000 but less than 5000 RWF	54	18.3	91.2
	Greater than 5000 RWF	26	8.8	100.0
	Total	295	100.0	

## Discussion

Community Health is seen as a holistic and integrated approach that takes into account the full involvement of communities in planning, implementation and evaluation processes, according to the Rwanda National Community Health Policy [10]. Furthermore, it is assumed to be an essential determinant of health and the indispensable ingredient for effective public health practice [8]. This study aimed to evaluate the availability of antimalarial medicines, inventory management at the decentralized community level. The findings indicated that CHWs are critical players in the management of antimalarial medicines at the community level. These findings are higher compared to the findings from Rwanda Demographic and Health Survey 2019–2020 which reported that among children with a fever for whom advice or treatment was sought, 17% received advice or treatment from community health workers [17].

### The availability and the rate of stock out of antimalarial medicines (for both young and adult doses) at the community level/among Community Health Workers

Ensuring availability of medicines is the ultimate goal of an inventory management system in public health facilities [18]. Medicines must be on hand when clients need them. This study found that 1,825 Artemether-lumefantrine (Coartem) drugs were consumed in November 2021 and the day of the visit, 78% of CHWs had minimum one packet of antimalarial medicines in their stock while the maximum was twelve. Medicines were considered available if it is not stock-out (if physical count is not zero) on the day of visit in the CHWs store [19].

This study reported 3.2% antimalarial stock out on the day of the visit. Compared to other African countries, Rwandan prevalence of antimalarial stock out is a bit lower as indicated by a study conducted in Uganda, which stated that ACTs/Coartem − 20/120 mg, a drug used to treat Malaria, was the least available (at 3.3%) of all the iCCM (Integrated Community Case Management) medicines [12]. A study conducted in Ethiopia, Malawi, and Rwanda as evidence for improving community health supply chains, indicated that in Malawi 27% of CHWs had 4 key iCCM products on day of visit. The same study indicated that in Rwanda 49% of CHWs had 5 key iCCM products on day of visit, whereas in Ethiopia 24% of CHWs had 5 tracer iCCM products in stock [20].

### Assessment of the status of antimalarial related inventory management practices, at community level, in Bugesera District in Rwanda

Findings showed that different methods were used to determine the quantities to order. For resupply, more than 50% of CHWs reported submitting requests when stock runs low or stocked out. The same findings showed that 29% of the CHWs ordered the quantity of antimalarial based on forms submitted and whenever the last container opened. Compared to 2014, this has improved as there was unstructured approach with no defined rules or processes to drive resupply as indicated in a study done from Ethiopia, Malawi, and Rwanda. In this research, findings revealed that 62% of CHWs resupplied based on (non–standard) documentation; 19% of CHWs used a variety of (“other”) methods; 8% of CHWs provided the same as previous month before the study; 7% and 4% of CHWs “didn’t know” or used a formula, respectively [19].

Storage being considered as a precondition for the main objective to ensure that the drugs maintain their quality and effectiveness to treat malaria [21], majority 78% of the CHWs have the appropriate storage. In assessing the storage situation, a list of key storage conditions was identified, and data collectors observed whether these conditions were individually met or not.

Respondents representing 21.36% of CHWs interviewed during data collection on these conditions, reported inappropriate storage space as a challenge. The findings corroborate with a systematic literature review done in Low- And Middle-Income Countries (LMICs) which reported storage challenges as reasons for stock-out among CHWs [19], limited or inadequate or improper storage space, which led to stock-outs among CHWs.

The issue of inadequate storage space is not unique to CHWs, who typically store supplies in a box in their homes [19]. The findings showed that 4.7% CHWs did not store securely with a lock and key and with limited access while 8.8% did not store antimalarial medicines in clean, dry, well-lit, and well-ventilated storeroom. Furthermore, 12.2% did not store medical products on shelves or stacked off the floor in stacks and away from walls.

### Prevailing challenges in inventory management practices of antimalarial medicines

CHWs often work in difficult conditions with little or no immediate technical assistance owing to poor recognition of their activities [22]. Some of the challenges that have impeded the smooth running of the work of CHWs include, but not limited to, lack of appropriate or dependable transportation and at a certain extent, inappropriate drugs storage.

This study discovered that, 25.4% traveled the distance between 1 and 2 h for resupply, 34.6% reported that they face challenge of both transport and inappropriate drugs storage. Respondents representing 31.1% reported that the only challenge they face is transport while 51.2% CHWs claimed to spend more than 2000 RWF per month from their own pocket on community health Duties. These results validated the study of Michelle D. S. Boakye aimed to identify challenges of achieving sustainable community health services. The findings of the study reported that the CHWs are most of the time challenged to travel far distances to attend to some of their clients. Occasionally, they are forced to use their own money to hire bicycle services that comes in the form of bicycle or motor bike “taxis” [23]. The study corroborated with the study conducted by Schurer et al. [24], to evaluate the opportunity costs and benefits among Community Health Workers in Rwanda. It reported that CHWs contributed approximately 18% of their income to costs associated with patient care (e.g. patient transportation).

For better management of the antimalarial medicines, most of the CHWs declare the issue that needs to be tackled is traveling fees [9], to avoid traveling long distances.

Motivated CHWs encourage the community to adopt practices that promote health. Appropriate trainings, encouragement, and rewards may help them perform better. This might involve providing identity clothes or badges, bicycles or transportation money, clear work descriptions, and performance evaluation systems [25].

### Study strengths and limitations

This study presents key findings related to the availability of antimalarial medicines, inventory management and related challenges at the community level in Rwanda. However, findings were self-reported by community health workers and may not truly represent the actual availability and stockout of antimalarial medicines at community level. Therefore, further studies are recommended using the validated WHO/Health Action International methodology.

It is advised that the further studies emphasize on the inventory management practices and supply chain performance at higher health facilities level. Appropriate transport facilities were also suggested to be given to CHWs to ease their work and avoid unnecessary expenses.

## Conclusion

This study showed that Community Health Workers help community to access quickly antimalarial medicines. Hence, more than 1,825 blisters of Artemether-lumefantrine (Coartem) drugs were consumed in one month. The study revealed that, 77.97% of the CHWs have appropriate storage conditions. CHWs disclosed that the lack of appropriate transportation is a critical barrier to accessing antimalarial medicines, where almost majority of them 70.85% reported that they travel on foot for resupply. This was reported as a challenge hindering their performance.

CHWs are capable and ready to serve the community, but Government has yet to create a pleasant working atmosphere for them [6]. Both regular and targeted training are necessary, to fill gaps in knowledge and must be in conjunction with sufficient follow-up and refresher courses. CHWs can perform well when they are well trained. However, they should be certified before being deployed, have refresher training, and be closely supervised after training to increase confidence in their performance [26].

### Electronic supplementary material

Below is the link to the electronic supplementary material.

**Supplementary Material 1:** Research Questionnaire

## Data Availability

The datasets used and analyzed during the current study are available from the corresponding author on reasonable request.

## Abbreviations

ACT: Artemisinin-based Combination Therapy
CHWs: Community Health Workers
HMIS: Health management information system
HSCM: Health Supply Chain Management
ICCM: Integrated Community Case Management
MOPDD: Malaria & Other Parasitic Diseases Division
RDT: Rapid Diagnostic Test
WHO: World Health Organization
