# Supplementary material for: Availability of antimalarial medicines and inventory management at the community level: a case study of Bugesera district in Rwanda
Source: BMC Health Serv Res. 2024 Jan 24;24:136. doi: 10.1186/s12913-024-10605-z (PMC10809492; doi:10.1186/s12913-024-10605-z)
Supplement: Supplementary file 1 — Supplementary Material 1: Research Questionnaire [file 12913_2024_10605_MOESM1_ESM.docx]

**RESEARCH QUESTIONNAIRE**

##### *This questionnaire/interview guide used in this study was developed for this study and not previously published elsewhere.*

##### Participants’ Consent Form Introduction

My name is Godelive Umulerwa Gakinahe, a Masters student at University of Rwanda at the EAC Regional Centre of Excellence for Vaccines, Immunization and Health Supply Chain Management. I am conducting a study on ***“*Availability of antimalarial medicines and inventory management at the community level: a case study of Bugesera district in Rwanda.*”*** You have been selected to take part in this study and should you decide to participate, confidentiality and privacy of the information you give will be assured.

##### Study purpose

The study focuses on the Availability of antimalarial medicines and inventory management at the community level: a case study of Bugesera district in Rwanda.

##### Study procedure

Participating in this study encompasses answering queries contained in the questionnaire. You are supposed to respond to the questions with guidance from the research assistants. At any particular period, feel free to query and seek further information on all the areas covered in this research.

##### Voluntary participation

This study is voluntary and you have the option to withdraw from the study at any given time. As well, you may decline to answer some questions which may interfere with your religious and cultural beliefs.

This is a detailed interview and may interfere with your daily routine duties. The interview may take about five to 10 minutes maximum. Despite these inconveniences, I will be grateful if you agree to participate in the current study.

##### Confidentiality

The provided information will be treated with utmost confidentiality and privacy. The researchers will not disclose your identity and the provided information will only be used for the purpose of this study.

##### Contact information

In case of any further clarification regarding this research, kindly feel free to contact me at the following contact:

Godelive Umulerwa Gakinahe

Email: gakinahe.godelieve@gmail.com

Tel No: 0788453363

**Participant’s statement**

The clarification regarding my participation in this study has been sought and given best to my knowledge. I have been given a chance to ask queries. It is optional and voluntary to get involved in this research. To my understanding, the given information will be privately and confidently kept. I may also opt withdraw my participation in this exercise at any time and the fact that I sign before completing the questionnaire, means that I have willingly accepted to be part of the research.

Names: …………………………..

Sign………………………………

Date………………………………

#####

##### Research questionnaire

**Topic: Availability of antimalarial medicines and inventory management at the community level: a case study of Bugesera district in Rwanda**

Participant number……………………… Interview Date…………………………

***Instructions:*** Please tick or insert the option that best fits your response

**Section A:** Socio-Demographic Characteristics of the study population

| **Socio-demographic characterizes of study participants.** | |
| --- | --- |
| Date of interview | DD/MM/YY: ……/……./……. |
| Participant code |  |
| Sector name |  |
| Cell name |  |
| Village name |  |
| Gender | Male (1) |
|  | Female (2) |
| Age group (years) | - 25-35 - 35-45 - 45-55 - Above 55 |
| Highest level of education completed? | Primary (1) |
|  | Primary (2) |
|  | Higher education (3) |
|  | Other (specify) (4) |

| **Section II.** Assess the availability of antimalarial products (for both young and adult doses) at the community level/among community health workers and the rate of stock out of antimalarial products at the community level /among community health workers. | | |
| --- | --- | --- |
| **Questions** |  | **Your answer** |
| Which products and materials do you use to treat Malaria? | RDT |  |
|  | Antimalarial drug(s) |  |
|  | Paracetamol or acetylsalicylic acid (aspirin) |  |
| What is an RDT? | Test Pregnancy |  |
|  | Test Malaria |  |
|  | Typhoid fever |  |

|  | Packaging per dose by bodyweight of patient | Total available |
| --- | --- | --- |
| Artemether-lumefantrine (Coartem) | 5 to < 15 kg |  |
| Artemether-lumefantrine (Coartem) | 15 to < 25 kg |  |
| Artemether-lumefantrine (Coartem) | 25 to < 35 kg |  |
| Artemether-lumefantrine (Coartem) | 35 kg and over |  |
| RTD |  |  |

|  | Packaging per dose by body weight of patient | Consumption in November 2021? |
| --- | --- | --- |
| Artemether-lumefantrine (Coartem) | 5 to < 15 kg |  |
| Artemether-lumefantrine (Coartem) | 15 to < 25 kg |  |
| Artemether-lumefantrine (Coartem) | 25 to < 35 kg |  |
| Artemether-lumefantrine (Coartem) | 35 kg and over |  |
| RTD |  |  |

|  | Packaging per dose by body weight of patient | Stockout in November 2021? |
| --- | --- | --- |
| Artemether-lumefantrine (Coartem) | 5 to < 15 kg |  |
| Artemether-lumefantrine (Coartem) | 15 to < 25 kg |  |
| Artemether-lumefantrine (Coartem) | 25 to < 35 kg |  |
| Artemether-lumefantrine (Coartem) | 35 kg and over |  |
| RTD |  |  |

| ***Appropriate medical storage at the community level*** | | |
| --- | --- | --- |
|  | **Proposed answer** | **Your answer** |
| Are medical products stored in a clean, dry, well-lit and well-ventilated Storeroom? | Yes |  |
|  | No |  |
|  | I am not sure |  |

| **Identify inventory management-related challenges in distribution, reporting, and transporting antimalarial products.** | | |
| --- | --- | --- |
| **Question** | **Proposed answer** | **Your answer** |
| Where do people most obtain their antimalarial products? | Health Center |  |
|  | Community Health Workers |  |
|  | At local leaders |  |
|  | Other (specify) |  |
| Which mode of transport do you use for travel for medical resupply? | On foot |  |
|  | Bicycle |  |
|  | Motorcycle |  |
|  | Car |  |
| Distance to the nearest health Center for medical resupply. | Less than 15 min (1) |  |
|  | 15-30 min (2) |  |
|  | 30-1hour (3) |  |
|  | 1-2hours (4) |  |
| Which amount of money do you spend on your own pocket per month for medical resupply. | Less than 2000 RWF |  |
|  | Greater than 2000 RWF |  |
|  | Greater than 2000 but less than 5000 RWF |  |
|  | Greater than 5000 RWF |  |
